# Drug target prioritization by perturbed gene expression and network information

**DOI:** 10.1038/srep17417

**Published:** 2015-11-30

**Authors:** Zerrin Isik, Christoph Baldow, Carlo Vittorio Cannistraci, Michael Schroeder

**Affiliations:** 1 Bioinformatics Group, Biotechnology Center (BIOTEC), Technische Universitat Dresden, Tatzberg 47-49, 01307 Dresden, Germany; 2 Computer Engineering Department, Dokuz Eylul University, Tinaztepe Kampusu, Buca, 35160 Izmir, Turkey; 3 Institute for Medical Informatics and Biometry, Technische Universitat Dresden, Blasewitzer 86, 01307 Dresden, Germany; 4 Biomedical Cybernetics Group, BIOTEC, Technische Universitat Dresden, Tatzberg 47-49, 01307 Dresden, Germany

## Abstract

Drugs bind to their target proteins, which interact with downstream effectors and ultimately perturb the transcriptome of a cancer cell. These perturbations reveal information about their source, i.e., drugs’ targets. Here, we investigate whether these perturbations and protein interaction networks can uncover drug targets and key pathways. We performed the first systematic analysis of over 500 drugs from the Connectivity Map. First, we show that the gene expression of drug targets is usually not significantly affected by the drug perturbation. Hence, expression changes after drug treatment on their own are not sufficient to identify drug targets. However, ranking of candidate drug targets by network topological measures prioritizes the targets. We introduce a novel measure, local radiality, which combines perturbed genes and functional interaction network information. The new measure outperforms other methods in target prioritization and proposes cancer-specific pathways from drugs to affected genes for the first time. Local radiality identifies more diverse targets with fewer neighbors and possibly less side effects.

In drug discovery, drug target identification is an important problem. Drugs interact with targets and off-targets, which trigger downstream signaling cascades causing perturbations in the cell’s transcriptome. The term “target” can refer either to proteins physically binding to the drug or to proteins that are only functionally related. Drug-induced perturbations have been uncovered at very large scale in the Connectivity Map (CMap) for 1300 compounds on four human cancer cell lines^1^. The CMap provides the opportunity to find similar phenotypes between a given gene profile and drugs. Thus, it facilitates an elucidation of the drugs’ modes of action and generation of new candidates for drug repurposing. A recent study used CMap drug profiles and revealed a high conservation of drug-induced transcriptional modules for multiple cell lines with limited expression of drug targets^2^. If a drug does not alter the expression of its target, but if it does alter the expression of other genes, then what is the relation of the target to these genes? A drug modulates the activity of a target protein, which subsequently regulates down-stream proteins. Protein-protein interaction (PPI) networks provide such down-stream relationships between targets and proteins by using physical contacts, genetic interactions and functional relationships.

In the last decade, drug target prediction and repositioning problems have become more attractive with the availability of phenotype and network data. Various network measures (e.g., centrality measures, random walk, shortest path, nearest neighbor etc.) were integrated with gene expression profiles to validate known drug targets or to identify essential proteins. A comprehensive review summarizes network related target identification and repositioning methods^3^. A recent study developed a kernel diffusion method that integrates gene expression and network data and identified known drug targets with 0.9 AUC^4^. Another consensus-based approach was evaluated on 30 different diseases and achieved AUC values over 0.9 AUC for the prediction of known disease targets^5^. It showed that local and global network measures can reveal potential drug targets, and thus an integrated model of measurements could achieve a better performance. A network flow approach integrated a PPI network, gene expression data and disease genes to identify effective drug targets for prostate cancer^6^. Another random walk-based study predicted drug target interactions by using similarity metrics for drugs and proteins in the construction of a drug-target network^7^. However the studies do not integrate any drug perturbation data into the prediction method.

The topological analysis of biological networks is also performed for a better understanding of complex cellular processes. Network centrality measures were used to identify essential nodes in various species’ interactomes^3^. In this direction, a study proposed a novel centrality measure that incorporates a PPI network and gene expression data to identify essential proteins in yeast^8^. A recent study investigated gene expression characteristics on cancer pathways and showed the effects of four network centrality measures to identify cancer treatment targets^9^. They also found different therapeutic targets by changing the network topology (pathway or PPI) and introduced tissue-specific data. Another publication showed that structurally similar drugs regulate topologically closer genes in PPI networks, i.e., protein products of such genes have a lower shortest path distance versus regulated genes of dissimilar drugs^10^.

The availability of gene expression phenotypes and interaction network data raises the following question: Can drug targets be identified from network information and expression alterations induced by a drug? It is hypothesized that a drug perturbation can be observed from differentially expressed (i.e., deregulated) genes that work on specific biological processes that develop the observed phenotypes^11^. Although post-transcriptional regulations on mRNAs might change the amount of the translated proteins, we could not consider this factor in the scope of our study due to the lack of large-scale protein data measured upon drug treatment. We hypothesize that deregulated genes are close to drug targets in terms of network topology. The proximity of deregulated genes to drug targets might be determined by identifying the shortest paths in a functional interaction network, i.e., a deregulated gene could be a direct interactor or a close neighbor of the altered target. Other questions follow from this hypothesis. Does a global or a local network feature give higher target prediction accuracy? And, does the target prediction performance depend on the definition of a target protein? To address these questions, a drug target prioritization method was developed by proposing a new network measure, *local radiality*, which integrates both topological data and the perturbed gene information. Radiality is a well-known centrality measure describing the level of node reachability of a node via different shortest paths of the network. The new measure describes the reachability of a target protein via the shortest paths to deregulated genes. Thus, it is a new locally constrained radiality. For simplicity, we will refer to this measure as *local radiality (LR)*. After investigation of recent studies^3^,^4^,^5^,^6^,^7^,^8^,^9^,^10^ and network centrality metrics^3^,^12^, we chose state-of-the-art methods^4^,^8^ and compiled a set of 13 measures (including LR) to assess their performance in target prediction problem. The goal was to construct a representative set of metrics, i.e., some metrics only use gene perturbation data, some benefit from the network topology, and others integrate both types of data by applying either shortest path-based or random walk methods.

The novelty of this study is the comprehensive evaluation of various measures that use the drug perturbation data and/or functional interaction networks for target prioritization. The LR measure achieved the highest prediction rate (22%) for known targets ranked in the 1^st^ percentile of all proteins from a functional interaction network. To the best of our knowledge, LR offers the best predictions compared to other methods in the field. The innovation of this method is not only the effective prioritizing of known targets but also the detection of less obvious or diverse targets in the biological network. Additionally, the cancer tissue-specific pathways are highlighted by extracting shortest paths between perturbed genes and known targets. These deregulated paths compose drug-target deregulated genes sub-network and might better explain an observed phenotype.

## Results

**Figure 1:**
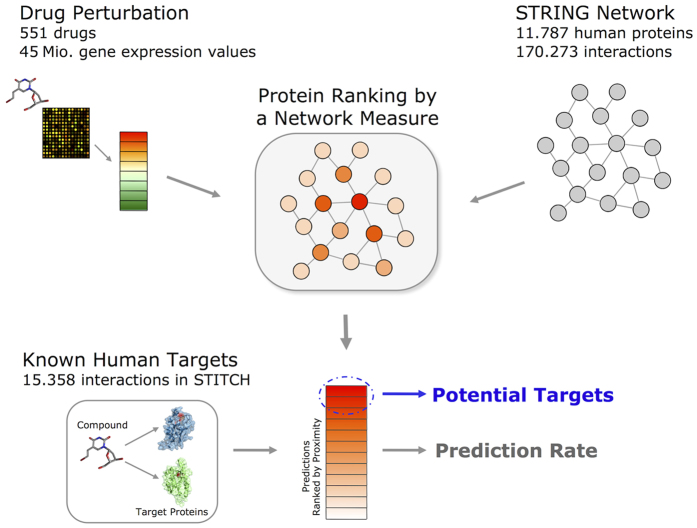
Overview of the target prioritization method. The perturbation of a drug on a specific tissue is measured by microarray experiments. Deregulated genes are obtained by comparison of drug-treated and control samples. A network measure computes a proximity score for each protein in the biological network based on its expression value, location to the deregulated genes or topological features. The proximity scores rank the possible drug targets, i.e., proteins with higher chance of being a target ranks on top of the sorted list. The target prioritization is evaluated by checking the rank of known drug targets (obtained from STITCH) in the sorted list of all proteins. The proteins listed in the high rank levels might be new potential targets.

Different network centrality measures and known target data are analyzed to observe their potential for drug target prioritization. Drug perturbation data is included in the calculation of topological proximity by using either deregulated genes or expression values themselves (Fig. 1). A centrality measure computes a closeness score for each protein by employing network topological features and expression values of deregulated genes. If a protein is not present in a PPI network, it cannot be predicted as a candidate target of a drug. The candidate targets are prioritized based on the closeness scores, i.e., proteins with a higher chance of being a target ranks on the top of the sorted list. Correlated with the initial hypothesis of proximity, a known drug target is expected to be at the top of the ranked list. To eliminate as many false positive target predictions as possible, only the proteins predicted in the 1^st^ percentile of the ranked list are suggested as potential drug targets. The proposed method was evaluated on the public CMap expression profiles.

### Gene expression is not sufficient for target prediction

**Figure 2:**
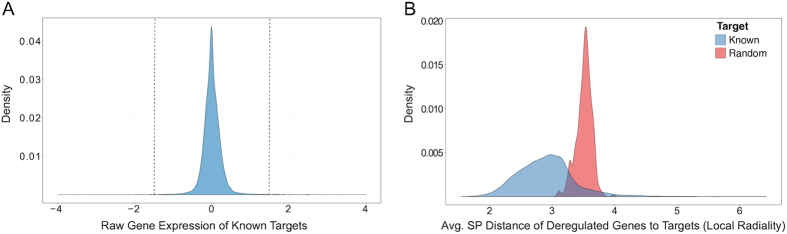
The distributions of drug targets. (**A**) Gene expression distribution of the 42331 known drug targets in the CMap. The significant targets reside on the right and left side of dashed lines. 97% of drug targets do not show significant expression changes due to drug perturbations. (**B**) The distribution of the average shortest path distances of deregulated genes to known (blue distribution) and to random (red distribution) targets. Two distributions are statistically different (Mann–Whitney, *p*-value < 2.2e−16). Deregulated genes are closer to known targets than any other proteins in the network. Thus, this motivates a network based target prediction.

Gene expression data represents mRNA activity of genes under a specific condition (i.e., control vs. drug treatment). In order to understand the capability of simple gene expression data in target prediction, the gene expression values (fold change—FC) of 42,331 known targets for the CMap drugs are analyzed (Fig. 2a). When significant targets are filtered (|FC| ≥ 1.5, *p*-value ≤ 0.05), 97% of all targets do not show any expression changes due to drug perturbations. A previous study also indicated the limited regulation of drug targets at the mRNA level^2^. Hence, gene expression data alone can predict only 3% of known targets.

### Network data improves the target prediction

Gene expression data can identify very few known drug targets. This begs the question: If a drug treatment does not change the expression of a target directly, could the target be predicted by the integration of other information? The utilization of PPI networks could enrich the gene expression data and therefore enhance target prediction. To illustrate the benefit of the network information, the distance of deregulated genes of each drug treatment to a potential drug target is calculated by taking the average of shortest path distances between each deregulated gene and target in the biological network (here, STRING) for CMap drug profiles (Fig. 2b). We selected 1000 different random targets (out of the biological network) for each known drug target. Then, the shortest path distances of deregulated genes to random targets and the known target are calculated separately. While the average distance of known targets to deregulated genes is 2.9 nodes, it is 3.6 nodes for randomly selected targets. Two distributions are statistically different (Mann–Whitney, *p*-value < 2.2e−16). This observation supports the initial hypothesis that deregulated genes are closer to known targets compared to most proteins in the network. Based on this observation, we formulated *LR* in the drug target space. This measure uses a set of deregulated genes *DG* and a biological network *G*. The *LR* score of node *n* in the network *G* is calculated as follows:


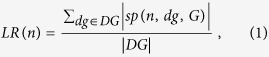


Here, the function *|sp|* calculates the length of a shortest path that connects the deregulated gene *dg* and the node *n* in *G*; *|DG|* indicates the total number of deregulated genes. The LR utilizes both drug perturbation data (i.e., deregulated genes) and topological information (i.e., shortest path distance). Thus, it implements the hypothesis about the proximity of deregulated genes to corresponding drug targets.

### Systematic Evaluation of 13 Measures

We also considered 13 different network measures to evaluate LR. Some are purely topological and others consider gene expression as well as various network interactions such as functional and physical interactions. The 13 measures are summarized in Supplementary Table 1. Two measures, FC and *p*-value, only consider gene expression data. We do not expect these measures to perform well because drug targets are generally not differentially expressed. The majority of measures, 11, use network information (4 in combination with expression, 7 without). The performance of these 7 measures will define a baseline showing how a general network without application-specific data performs. Of the 11 network-based measures and considering network topology entirely, 7 are local and 4 are global. Five network measures are based on the shortest path and 2 on the random walk approach.

### LR performs best

**Figure 3:**
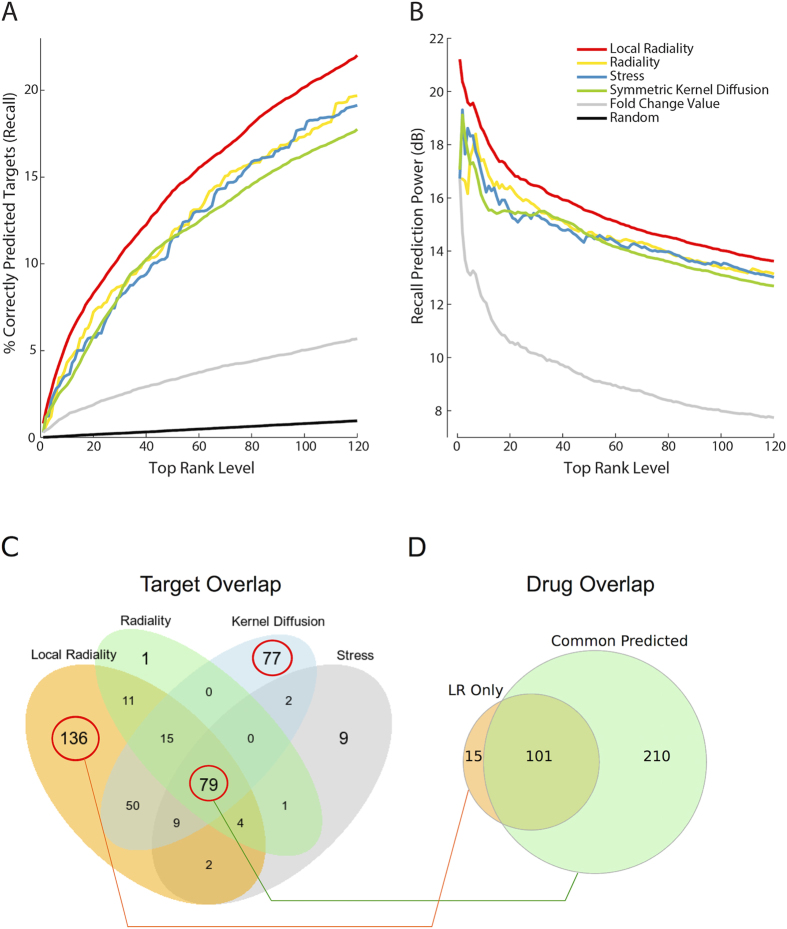
Results for drug-target prioritization methods. (**A**) Prediction performance of the selected measures for functional targets (FT). The y-axis shows the cumulative percentage of correctly predicted targets (i.e., recall) of all drugs in the CMap, the x-axis gives the predicted rank level. The predictions are given for the 1^st^ percentile (top 120) of the ranking list. The LR achieved 22% recall value, which is the highest prediction rate. (**B**) The prediction power (expressed in decibel, dB) of each measure compared to the random predictor. It shows the magnitude of recall for each predictor normalized with respect to the random predictor. (**C**) The overlap of known targets that are predicted in the 1^st^ percentile. 79 targets (common predicted) are predicted by all measures. Radiality and stress usually predict similar targets, however LR (136 unique targets) and kernel diffusion (77 unique targets) predict different ones. (**D**) The overlap of the drugs that bind to proteins found by only LR (LR Only) and all measures (Common Predicted). There were 331 different drugs that bind to 79 proteins, which are predicted by several measures. However, 15 drugs bind to specific proteins that are predicted only by LR. In other words, common targets are usually well-studied proteins, while the LR targets are more specific ones and have more potential for new drugs.

LR is the only measure combining expression and local network data based on shortest paths. We systematically compared all 13 measures (see Supplementary Fig. 2 for all measures). The performance of best predictors is shown in Fig. 3. Predictions based only on FC values are also included for better comparison. The LR performs best overall under all configurations. The random predictor ranges from 0.1 to 1% (see Fig. 3a). The LR predicts up to 22% of targets in its 1^st^ percentile of predictions. Fig. 3b shows the performance of selected measures relative to a random predictor (*prediction power*). The symmetric kernel diffusion ranking is a successful metric developed recently^4^. Its recall value (0.17) is significantly (Wilcoxon signed rank test, *p*-value < 1.7e−16, Supplementary Table 3) lower than LR (0.22). The result is similar for the AUC value; the symmetric kernel diffusion ranking has an overall 0.81 AUC, and LR has 0.85 AUC (Supplementary Fig. 3a). The PeC was developed as the essential protein discovery measure^8^, and LR has a significantly (*p*-value < 5.7e−24) higher recall value versus PeC (0.17). PeC has a lower AUC (0.82) compared to LR (0.85). A recent study applied degree and betweenness to identify cancer targets^9^. In terms of recall values, LR significantly outperforms both degree (*p*-value < 1.2e−44) and betweenness (*p*-value < 2.7e−16). Similarly, LR has a higher AUC value compared to degree (0.81 AUC) and betweenness (0.80 AUC). In summary, LR performs significantly better (average *p*-value < 5.3e−06, Supplementary Table 3) than other predictors in the 1^st^ percentile. The overall performance of LR also outperforms others with an AUC value of 0.85 (Supplementary Fig. 3a).

### Gene expression vs. network data

In the previous sections, we argued that gene expression is not sufficient for target prediction and that network data could improve it. Figure 3a supports this hypothesis: FC achieves only 3% correct prediction versus 22% for LR. Furthermore, all network-based measures shown in Fig. 3a all perform much better than FC (Wilcoxon signed rank test, average *p*-value < 1.3e−42). Thus, we conclude that network data is crucial in target prediction.

### Network-based measures are the top predictors

The best predictors—LR, radiality, stress, and symmetric kernel diffusion—use the network topology for the calculation of a node score. Radiality indicates the level of reachability of a node via the shortest paths to all other nodes (i.e., the closer to the rest of all nodes, the easier it is to reach). Stress calculates the frequency of a node to appear in all possible pairwise shortest paths of the network. Although radiality (0.20 recall, 0.83 AUC) and stress (0.19 recall, 0.81 AUC) only use network topology, they perform quite well versus the FC-dependent measurements. Symmetric kernel diffusion is a random walk-based method, which applies gene expression data as initial node scores. It achieved 0.17 recall for the 1^st^ percentile and overall 0.81 AUC.

### LR predicts twice as many different targets than other measures

Gene expression independent measures (i.e., radiality, stress) achieve quite high prediction rates, however they usually predict the same targets for the 1^st^ percentile in contrast to gene expression dependent ones (e.g., LR, kernel diffusion). All four measures agree on the 79 targets. In contrast, 136 and 77 target proteins are predicted with only LR and kernel diffusion, respectively (Fig. 3c). When only LR and kernel diffusion predictions are considered both methods predict the same 153 targets in their 1^st^ percentile. On the other hand, the proportion of predictions exclusively obtained by LR and kernel diffusion respectively is 153/79 (=1,9). Thus, more than twice the number of known targets are predicted by LR versus kernel diffusion.

### LR targets have fewer drugs and fewer interaction partners

The 79 common proteins indicated in Fig. 3c are targeted by 311 drugs (see Fig. 3d). On the other hand, 136 proteins predicted only by LR have only 15 drugs (Fig. 3d). This suggests that the common targets are well studied, while the LR targets are more specific and have more potential for new drug findings^9^.

**Figure 4:**
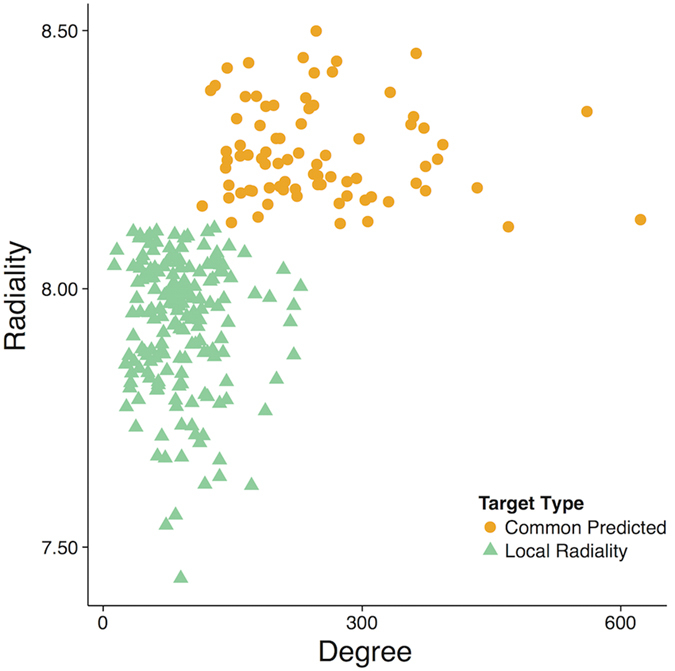
Topological characteristics of frequently predicted target classes: only *LR* (green triangles), *common predicted* (orange circles). The average degree of the known targets identified exclusively by LR is 94. For the common predicted targets, it is significantly larger (σ = 248). Similarly, the average radiality of targets identified by LR is relatively small versus the common predicted ones. These facts indicate that LR detects the targets, which represent hubs in local network modules rather than in the entire network.

Degree and radiality are two key network features that explain the behavior of drug targets in terms of network topology (Fig. 4). Common predicted targets (orange circle) have both a high degree and high radiality values versus the targets predicted by only LR (green triangle). This observation suggests that the commonly predicted targets are well-connected proteins in terms of neighbors and shortest paths. Hence, such topological features make them easily predictable. Conversely, LR can predict less obvious drug targets by integrating gene expression data and topological information. We speculate that such targets might lead to fewer side effects and provide more effective treatment results by having fewer neighbors in the network.

### Functional targets are more favorable for target prediction

There are several ways to define a drug target. Is the drug physically binding to the target or is there a indirect functional relationship? Are all known targets of a drug considered or only those of highest confidence? In order to evaluate the impact of different drug target definitions on our analysis, we created three drug-target sets:

-Physical targets (***PT***) are collected from 15 different drugs, proteins, and compound databases (see the Drug Targets Section).

-Functional targets (***FT**, **FT1***) are obtained from the STITCH Database^13^.

In Supplementary Table 2, all target sets cover around 500 drugs. However they differ strongly in the number of targets. FT1 is a subset of the FT targets, it only considers the most confident target for each drug (according to literature based search on PubMed). Hence FT has many more targets (2782) than FT1 (195). The physical target (PT) set contains 605 targets.

**Figure 5:**
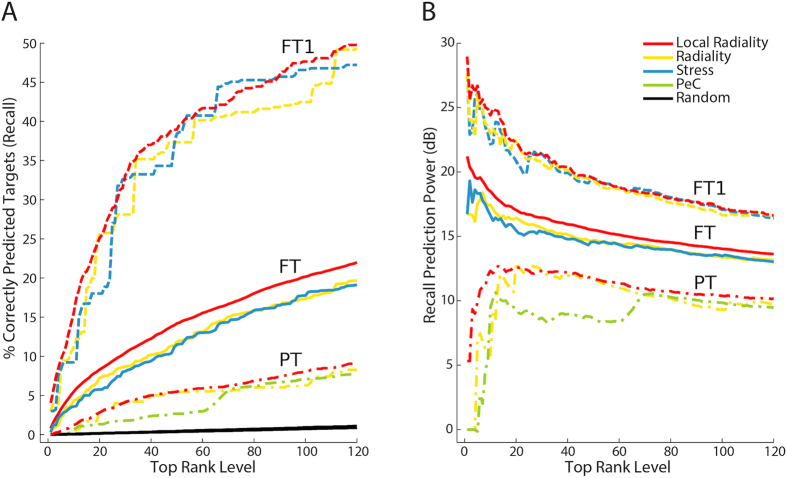
Results for different drug target data sets. (**A**) Comparison of functional (FT, FT1) and physical (PT) targets for selected measures. The predictions are given only for the 1^st^ percentile of the ranking list. Note that due to very close recall values, three random predictor curves are over plotted. The highest recall (50%) was obtained on the FT1 (limited functional targets). Half of the measures correctly predicted 15% to 22% of the FT (all functional targets). The recall values are between 5% and 9% for PT (physical targets). Although the performance of the measures is highly dependent on the target definition, LR achieved the highest recall values for all target definitions. (**B**) The prediction power (expressed in decibel, dB) of each measure compared to the random predictor. It shows the magnitude of recall for each predictor normalized with respect to the random predictor.

Figure 5 shows the performance of the 3 best measures on different target sets (see Supplementary Fig. 2 for all measures). The performance of these measures is highly dependent on the target set. Half of the measures correctly predict 15% to 22% of the FT set (Fig. 5a). In the PT set, the performance of the majority of the measures is between 5% and 9%. LR achieved the best prediction with 0.09 recall (average *p*-value < 1.2e−2, Supplementary Table 5) for the 1^st^ percentile; overall AUC value was 0.76 (Supplementary Fig. 3c). The PT set represents physical drug target interactions, and their performance is much worse than the performance based on the FT set. One reason might be that the STRING network is more adequate for the identification of functional targets, which were obtained by text mining methods. The highest prediction rate is achieved on the FT1 set—the best ones predict 50% of known targets. LR and radiality achieved 0.497 and 0.493 recall (Supplementary Table 4) and overall AUC values of 0.929 and 0.924 (Supplementary Fig. 3b), respectively. Such a significant improvement is reasonable because text mining methods select well-studied genes with many literature references as targets that generally have many connections versus other candidate targets in the STRING network. 82% of all targets in FT1 have a degree higher than 50 (Supplementary Fig. 4). This proves the high connectivity of the targets, which are true-positives in many cases.

In general, the functional target sets (FT, FT1) outperform the physical targets (PT). The recall and AUC in the physical target prediction are low versus the functional ones because the known physical targets are limited due to experimental difficulties in the identification. However, the prediction of the physical targets performs better than the random one (Fig. 5b). The PPIs in the STRING network are useful in predicting the functional relations between drugs and targets provided by the STITCH database, but this is not that much efficient in predicting the physical interactions due to incomplete target knowledge of the PT. Note, LR performed equally well for all target definitions that support its prediction capability. In conclusion, drug target prediction is strictly dependent on the targets chosen for validation purposes.

### A Sub-network of Selected Targets and Deregulated Genes

**Figure 6:**
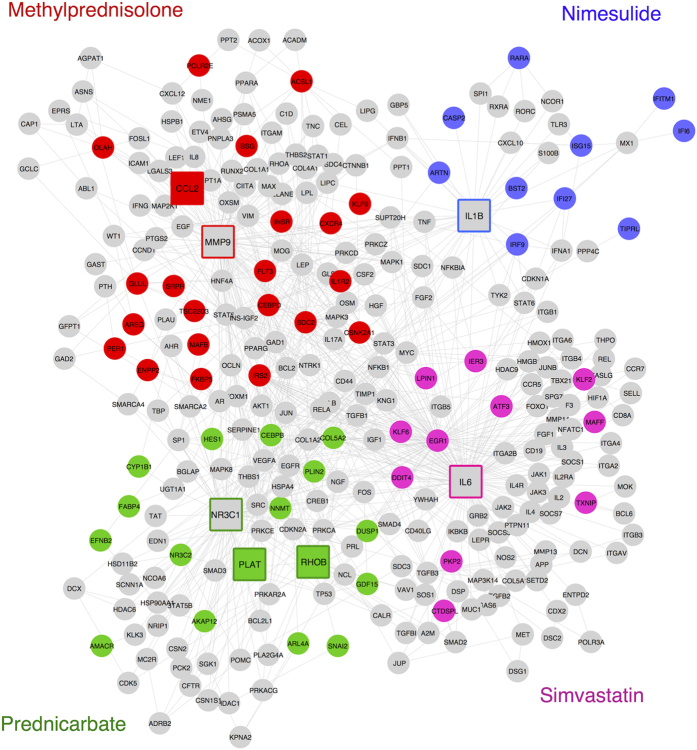
A sub-network of selected targets and deregulated genes. Four drugs (methylprednisolone, nimesulide, prednicarbate, and simvastatin) and their differentially expressed genes are shown in different colors in the STRING network. A rectangle node shape represents a target protein, and circles indicate interconnecting genes. Differentially expressed genes (including possible targets) are colored in the color of the appropriate drug. Therefore, each colored sub-network might represent affected downstream pathways of the given drug. Thus, the view of target-affected genes community helps experimentalists design new drug experiments.

Although the prediction of a drug target is crucial, the generation of the expected phenotype is also important for drug treatment experiments. The pathway databases can help to formalize the expected phenotype, but incomplete databases limit the investigation of effects on the pathway level. Specifically, the knowledge about molecular pathways might be incomplete and inconsistent between different sources^14^ because biochemical reactions are not fully understood for all genes and diseases. If such information is not covered for a drug target and the affected genes in public databases, the PPI networks might provide some hints for possible reactions between these genes. Therefore, we used the LR method to obtain more insights into affected downstream pathways (see details in the Methods Section). The extraction of shortest paths between deregulated genes and known targets exposes the topological mapping of perturbation data in a functional interaction network (Fig. 6). Each selected target-deregulated gene sub-network is clearly separated from other nodes in this example. Each colored sub-network might be interpreted as affected downstream pathways of the given drug. Such deregulated paths explain the observed phenotype after a drug treatment. Moreover, the *sub-network of selected targets-deregulated genes* might point out potential new targets for the given drug. Thus, this network-level visualization helps experimentalists design new drug experiments.

### Downstream Affected Pathways of Targets

The LR shows the proximity of deregulated genes to targets. It also identifies the affected pathways. Therefore such paths are extracted by the paths passing through the known target(s) and deregulated genes (see details in the Methods Section).

**Figure 7:**
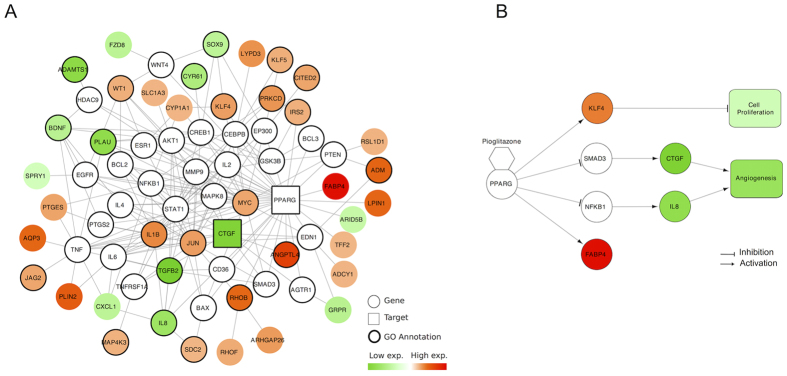
Downstream affected pathways for the Pioglitazone treatment. (**A**) The shortest paths network. The colored nodes represent deregulated genes and bold circled nodes have specific Gene Ontology annotations (e.g., angiogenesis, apoptosis). (**B**) The core pathway affected by the activation of PPARG. The color indicates the gene expression value of the node. An edge represents an activation or inhibition between two genes.

In particular, we investigated Pioglitazone as well as its targets and altered pathways. Pioglitazone was approved for the treatment of type 2 diabetes. It regulates the peroxisome proliferator-activated receptor gamma (PPARG) as an agonist. Connective tissue growth factor (CTGF) is reported as a functional target of Pioglitazone in the STITCH database. CTGF is involved in endothelial cell proliferation, migration, and angiogenesis. Several network measures ranked PPARG and CTGF in the 1^st^ percentile of possible targets on the prostate cancer (PC3) tissue. Thus, Pioglitazone might be a new repositioning candidate for prostate cancer treatment. Although high expression of CTGF was observed in tumor-promoting prostate stromal cell lines^15^, it is significantly down-regulated by the Pioglitazone treatment; thus it can no longer trigger the angiogenesis path. The affected pathways due to the Pioglitazone treatment were analyzed using the LR measure. There were 70 deregulated genes in this treatment with |FC| ≥ 2. The shortest paths network (SP-net) is built by compiling the shortest paths passing through PPARG, CTGF (targets) and deregulated genes based on the STRING network. The initial SP-net contains 322 genes and 1125 edges. Figure 7a shows possible affected paths after application of a filtering procedure (see Methods). The most interesting genes are SMAD3, NFKB1, IL8, KLF4, and FABP4. We performed a literature search to find transcriptome-level responses of these genes. PPARG agonists inhibit CTGF expression through SMAD3-(4)^16^. Similarly, PPARG agonists reduce SMAD3 activity and inhibit metastasis of lung cancer cells in mice^17^. Due to the down-regulation of CTGF through SMAD3 inhibition, these observations could be accurate for Pioglitazone treatment on PC3 tissue. Activation of PPARG represses the transcriptional activity of NFKB that reduces IL8 production and proliferation of PC3 cells^18^. A similar mode of action might work in the Pioglitazone treatment because of the down-regulation of IL8. Epidermis-associated FABP is strongly down-regulated in prostate cancer cells^19^,^20^. Correlated with such an observation, PPARG activation leads to a significant up-regulation of FABP4 in the Pioglitazone treatment. KLF4 regulates cell proliferation, apoptosis, and inflammation. KLF4 works as a tumor suppressor^21^ and PPARG binds to the promoter region of KLF4 in prostate cancer^22^. The up-regulation of KLF4 in the Pioglitazone treatment also supports previous findings that it might reduce tumor proliferation. All of these observations, which are obtained by the affected pathway analysis and validated by pathway databases (e.g., KEGG, Reactome, and WikiPathways), uncovered the hypothetical pathway in Fig. 7b. In summary, previous studies highlighting the relationship between PPARG and CTGF, IL8, and KLF4 were also confirmed by the affected pathway analysis, which helps in the discovery of a pathway-level phenotype for drug treatment.

## Conclusion

This study integrates gene expression profiles and protein-protein interactions to prioritize possible drug targets. One of the essential factors in the prediction quality is the network measure, which is used for protein scoring. The novel measure, LR, achieves the highest target prediction rate versus previous studies—it can predict 22% of the known targets in the 1^st^ percentile. Additionally, it is more promising for predicting diverse drug targets. The STRING network, which integrates various PPI networks and predicted interactions, accomplishes the best performance together with the STITCH target set. This might be because of the construction schema because some interactions in both databases are obtained by literature mining and prediction methods. Hence, well-studied targets have a tendency to be highly connected within the biological network. The selection of high-degree nodes as drug targets might have a toxic effect on patients^9^. Toxicity might also appear by regulating highly central genes in the network^10^. On the other hand, only about 20% of the estimated human interactome is currently known^23^, thus, such a sparse network might also limit the prediction capability of the method. Therefore, the performance depends markedly on the selected network measure as well as the definition of a target protein. In conclusion, the integration of gene expression data into biological networks improves the prioritization of known drug targets. The shortest path-based approach, LR, uncovers affected pathways due to a drug perturbation. Thus such affected pathways explain the observed phenotype. Moreover, a sub-network of selected targets and deregulated genes highlight potential new targets for the given drug. Furthermore, the predicted targets in the top-ranked positions might be used as an input for docking algorithms that can compute the likelihood of a physical interaction with the given compound and candidate targets. Due to the high time consumption of docking algorithms, the proposed approach would dramatically reduce the amount of candidate proteins for further validation in binding assays.

Use of more comprehensive molecular interaction data, the integration of pathway information and tissue-specificity into a global interactome are possible future directions to build dynamic networks. We plan to update the human interaction network via online services such as PSICQUIC^24^. The pathway information provides the biological signal flow between proteins and the corresponding processes. Directionality can be obtained from pathway databases (e.g., KEGG, Reactome) or be inferred by searching for the shortest paths between specific receptors and down-stream affected genes^25^. Edge weights in a network might be defined by integrating gene expression correlations^6^,^26^. Considering edge directions and weights in such networks could improve the prediction capability of network measures. The global human interactome covers all possible interactions that may occur in different cell compartments, tissues, or experimental conditions. One approach for customizing the global interactome as a tissue-specific network might be the generation of interactions by considering co-expressed interaction partners in a specific tissue or condition^9^,^27^,^28^,^29^. Such a tissue–specific and weighted network could improve the identification of targets and downstream pathways.

## Methods

### Microarray Data Processing

Connectivity Map (CMap) was downloaded, and then raw microarray data of the control and the drug treatment samples were analyzed with the affy R-package (version 2.15.3). The CMap (version 2) contains 6100 microarray experiments showing treatment responses of 1309 drugs on cell lines MCF7, PC3, HL60, and SKMEL5^1^. The CMap is a well-established, comprehensive and widely used repository. Hence, it was chosen as an extensive drug perturbation data. The raw data were analyzed by the RMA method^30^, as provided in the affy R-package. The differential expression of a gene is represented by the fold change (FC), i.e., ratio of drug-treated versus control samples. Genes and nodes in networks are represented by Entrez gene identifiers, and thus probes with unknown Entrez identifiers were discarded. If a gene is represented by multiple probe sets, the probe set with the highest mean expression was selected as the representative. If the absolute FC value of a gene is higher than 1.5 (p-value ≤ 0.05), it is considered as a deregulated gene in the CMap data set.

### Interaction Network

Human protein interactions were obtained from the STRING database (version 9.0) and filtered based on the confidence score, which was computed during the integration of various data sources in STRING^31^. In order to limit the false–positive interactions – which are probably originated from prediction methods – interactions having a confidence score of 800 or above were kept. The resulting STRING network contains 11787 nodes (proteins) and 170273 edges (interactions) and represents 11% of all interactions in the original STRING network. The edges are used unweighted and undirected.

### Drug Targets

The known human targets of the drugs in the CMap database were extracted in several steps. First, each drug was mapped to its corresponding PubChem identifier based on a drug name comparison. Known human targets of these drugs were extracted from the STITCH database (version 3.1)^13^. In the STITCH database, drug-target interaction data are collected from different data sources, which provide information about metabolic pathways, crystal structures, binding experiments and drug target relationships. Afterwards, for every drug-target interaction, the likelihood of all different sources of this interaction was combined to achieve an overall confidence score. Drug-target interactions were extracted with PubChem identifiers from STITCH. Finally, human targets with a confidence score of 800 or above were selected as drug targets. This target set is called **FT**. After the mapping and filtering steps, 551 drugs with known targets were left in the CMap data set. Due to the high number of targets for some drugs, the most likely target of each drug was chosen with a text mining approach. The likelihood of being the best drug target is calculated based on the pairwise occurrence frequency of a target and a tissue name in PubMed abstracts (i.e., the more frequent, the more probable target it is). The most confident target shows a literature-based correlation with a specific tissue. Thus, the method selects tissue-specific targets. This target set, **FT1**, assigns only one target to each drug. **PT** (physical targets) is an in-house database aggregating more than 15 different drug, protein, and compound databases^32^. The PT set includes physical interactions from Protein Data Bank^33^, Therapeutic Targets Database^34^, and BindingDB Database^35^. The coverage of PT is much lower than of FT because of its focus on physical binding only. FT1 is the smallest target set in terms of unique targets and drug-target interactions (Supplementary Table 2). Although PT contains only known physical binding partners of queried drugs, it was successfully applied in previous drug repositioning studies^32^,^36^. The biological network (STRING) contains at least 90% of known targets that are provided by any target data set. The known target overlap between three drug-target data sets shows that 87 targets are indicated by all of the data sets (Supplementary Fig. 1). PT covers only 155 unique physical drug targets; 2224 targets are only provided by FT, and it has the highest coverage in terms of known targets.

### Network Measures

Several network measures were implemented, and the LR measure was developed for comparison purposes (Supplementary Table 1). Expression-specific measures integrate gene expression into their calculation. The shortest path-based measures integrate the shortest path distances into their formula. Kernel and correlation diffusion rankings are representatives for random walk-based algorithms, which use the entire gene expression data. If a measure is local, it only considers a node and its close neighborhood to determine the score. A global measure potentially uses the entire network topology to calculate the node score. The random target predictor ranks the proteins by using 100 random protein selections out of all nodes in the network. Hence, it provides a baseline for other measures. The PeC, kernel and correlation diffusion ranking measures were selected from previous studies for comparison purposes^4^,^8^. All measures are implemented in the R-Bioconductor environment (version 2.15.3).

The LR measure helps to prove the initial hypothesis that deregulated genes might be close to drug targets in terms of the network topology. It uses a network *G* and a set of deregulated genes *DG* as input. A score of a node *n* in the network *G* is calculated as defined in the Equation 1. If the network is unweighted, *|sp|* shows the minimum number of nodes to connect *dg* and *n*. *LR* utilizes both drug perturbation data (i.e., deregulated genes) and topological information (i.e., shortest path distance).

The following measures are well-known network centrality measures and only consider network topology. Stress calculates the frequency of a node in any shortest path of the network.


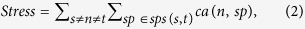


where *sps(s, t)* shows the set of all shortest paths from *s* to *t;* and *ca(n,sp)* is defined as follows:


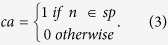


Radiality shows the level of reachability of a node via different shortest paths of the network (i.e., the closer to the rest of nodes, the easier it is to reach).


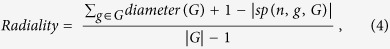


where *diameter(G)* indicates the length of longest path in *G*.

The formulation of the rest of measures is given in the Supplementary. The code of all measures and data sources (pre-processed CMap data, PPI network, drug-target data sets) are available under the http://projects.biotec.tu-dresden.de/DrugTargetPrioritization/ web page.

### Target Prioritization

Gene expression and network topological data are integrated to predict all possible drug targets. Drug perturbation data (i.e., control vs. treatment) is incorporated into calculation of the topological proximity by using either gene expression values or deregulated genes. The possible targets of a given drug are predicted by a sorted list according to the closeness scores. Target prediction aims to eliminate as many false positive target predictions as possible. Hence, the proteins predicted in the 1^st^ percentile of the rank list are the most probable drug targets. If a known drug target is ranked in the 1^st^ percentile of all possible targets, this prediction is accepted as a true positive one. The overall performance is provided by the accumulative percentage of correctly predicted targets of all drugs in the given percentile^37^.


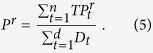


Here, *P*^*r*^ is the percentage of correctly predicted targets up to a rank level *r*, *TP*^*r*^_*t*_ is the number of true positive targets (which are predicted above the rank level *r*) of a drug *t*, *D*_*t*_ is the total number of known targets of the drug *t*, and *n* is the total number of drugs in the CMap. The *P*^*r*^ value corresponds to the true positive rate or recall value. Note, that each target has multiple rank values that are derived from different cell lines or tissues. To unify them into one rank value, the best rank value is chosen out of all predicted ranks. The percentage of correctly predicted targets is shown by a curve in which each point represents a *P*^*r*^ value for a specific rank level *r*. Thus, the x-axis represents the number of considered targets as positive predictions. That is, as more move to the right, target prediction becomes less precise. The y-axis shows the cumulative amount of correctly predicted targets (i.e., recall). To illustrate the deviation of each network measure with respect to the random prediction, we used *prediction power* transformation as described in the original study^38^.


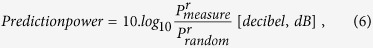


where *P*^*r*^_*measure*_ and *P*^*r*^_*random*_ are the recall of any network measure and random predictor, respectively.

We applied two groups of Wilcoxon signed rank testing to show that one measure predicts known targets better than the other. In this test, the recall curve of a measure is compared to another one. The distribution of the correctly predicted targets is assumed to be the same for all measures.

### ROC Analysis

Receiver Operator Characteristic (ROC) and AUC (Area Under the Curve) calculations compare performance of LR with previous studies^39^. Therefore we applied the same scheme as Laenen *et al.* to define TP (true positive), FN (false negative), FP (false positive), and TN (true negative) predictions^4^. We calculated true positive rate (TPR) and false positive rate (FPR) by applying all possible rank cutoffs (i.e., 1 to 11787) for the prioritization list. For each cutoff, we divide predictions into true and negative sets. Based on these sets, we defined TPs as all correctly-predicted known targets above or equal to the rank cutoff. FPs are all proteins ranked above, which are not in the known target set. FNs are known drug targets that are ranked below the cutoff. All remaining proteins are defined as TNs. Note, that definitions of TN and FP predictions should be taken with caution because these proteins might be known targets of new drugs in near future and thus incomplete target knowledge might lead a bias about definitions of the current scheme. The TPR and FPR of different rank cutoffs are used to plot the ROC curve, and finally an AUC value is calculated. The AUC value shows the probability that a randomly selected *known* target (positive one) is ranked higher than a randomly selected protein (negative one). Therefore an AUC value of 1 means that all known drug targets are ranked in the 1^st^ position of prioritization list, whereas a method with an AUC value of 0.5 ranks the target proteins not better than random chance.

### Construction of Sub-network of Selected Targets and Deregulated Genes

The sub-network of drug targets and deregulated genes show that the individual modules are composed of drug targets and deregulated genes. The aim is to extract the paths that pass through a target as well as the affected deregulated genes in the STRING network. Topological mapping of perturbation data in the biological network reveals the shortest paths between deregulated genes and known targets. To choose four drug examples given in the “A Sub-network of Selected Targets and Deregulated Genes” section, we applied the following selection scheme: The shortest paths network (SP-net) is extracted for each drug target and its deregulated genes. The SP-net^t^ is composed of all possible shortest paths that connect all deregulated genes and target *t*. *LR(t)* is the local radiality of a target *t* with the deregulated genes *dg*. It is calculated for each SP-net. If *LR(t) < 3*, and the distance of SP-net^t^ to other SP-nets is larger than 3, then SP-net^t^ is selected as an example for target-deregulated genes sub-network. Each selected SP-net^t^ is uploaded to the Cytoscape tool to visualize the sub-network^40^.

### Extraction of Downstream Affected Pathways of Targets

The aim is to extract the paths that pass through a target and the corresponding deregulated genes in the STRING network. If the length of a shortest path that connects a deregulated gene *g* and a target *t* is *3*, then all possible shortest paths with a length of 3 are counted for gene *g*. The SP-net of each target protein is constructed by using the same procedure as before. To focus on more specific paths, the SP-net is filtered based on two criteria: reaching the target within a fixed length of paths and selecting nodes with specific Gene Ontology (GO) annotations (e.g., angiogenesis, apoptosis). The fixed length is generally assigned to 2 because this SP-net covers direct interactors of deregulated genes and their neighbors. This provides a better consideration of the global topology. The rest of the analysis was performed on a limited size of SP-net with Cytoscape.

## Additional Information

**How to cite this article**: Isik, Z. *et al.* Drug target prioritization by perturbed gene expression and network information. *Sci. Rep.* **5**, 17417; doi: 10.1038/srep17417 (2015).

## Supplementary Material

Supplementary Information
