# Supplementary material for: Drug target prioritization by perturbed gene expression and network information
Source: Sci Rep. 2015 Nov 30;5:17417. doi: 10.1038/srep17417 (PMC4663505; doi:10.1038/srep17417)
Supplement: Supplementary Information [file srep17417-s1.pdf]

Drug target prioritization by perturbed gene expression and network information

Zerrin Isik, Christoph Baldow, Carlo Vittorio Cannistraci and Michael Schroeder

Supplementary Information

1 Definition of Measures

Fold Change

The fold change measure is constructed from the gene expression profiles as follows:

$$fc = log_2(\frac{dt}{t}),$$

where dt and t are the expression values for drug treated tissue and not-treated tissue, respectively. For every gene a fold change is provided and used as a score. Thus, the set $FC = (g, fc_g)$ where g is the gene identifier and fc_g (i.e. the fold change value of gene g) is directly used for ranking.

p-value of Fold Change

The p-value is computed by using the Student t-test for paired samples. Assume there is a drug sample d and a set of control samples C, X_D is defined as the set of differences between d and C. Then the p-value is calculated as follows:

$$p\text{-value} = \frac{\overline{X}_D}{\sigma_{X_D}/\sqrt{n}},$$

where σ_{X_D} is the standard deviation of X_D, n is the sample size. The p-value is providing whether the average of the difference is significantly different from d. Similar to the fold change a set, $FCP = (g, fcp_g)$ where g is a gene id and fcp_g (i.e. the corresponding p-value) is used as a score to rank all genes.

PeC

The PeC measure is proposed in another study [1], which underlies the combination of different terms. First, a highly connected protein is more likely to be essential, than a low connected one. Second, essential proteins in the same cluster are more likely to be differentially expressed and finally, essential proteins tend

to form densely connected networks, due to their high connectivity. The PeC measure considers these three assumptions and calculates first the edge clustering coefficient:

$$ECC(u, v) = \frac{|N_u \cap N_v| + 1}{\min\{d_u, d_v\}},$$

where N_u (or N_v) is the set of neighbors of gene u (or v) and d_u (or d_v) are the degree of the corresponding gene. The ECC is a local value and represents the likelihood of two nodes to be in the same cluster. The second component of the PeC measure is the Pearson correlation coefficient (PCC), which takes the change in the expression value into account, i.e. evaluation of co-expression of two proteins. CMap contains one sample for each drug treatment. Therefore the single drug treatment sample led us to use the inverse product of FC values instead of classic PCC. Nevertheless, PCC was changed into IP as the following:

$$IP(g_x, g_y) = 1 - \frac{1}{FC(g_x) * FC(g_y)}.$$

From that point the probability that two proteins are clustered together is considered from a topological and from a biological point of view. The overall probability of clustering two proteins g_1 and g_2 in one cluster is defined as:

$$P(g_1, g_2) = ECC(g_1, g_2) \times IP(g_1, g_2).$$

The PeC score for a gene g is defined as:

$$PeC(g_1) = \sum_{g_2 \in N_{g_1}} P(g1, g2),$$

where N_{g_1} shows the all neighbors of protein g_1.

Kernel Diffusion Ranking

The kernel diffusion ranking score uses the gene expression value of a node and neighborhood information, the method originally was developed by Laenen *et al.* [2]. It applies a random walk approach, but is based on a Laplacian matrix. Two different versions, an asymmetric and a symmetric one, are constructed that differ in the Laplacian matrix. The asymmetric, normalized version is defined as:

$$L_a = I - D^{-1}A.$$

The symmetric Laplacian matrix is defined as:

$$L_s = I - D^{-\frac{1}{2}}AD^{-\frac{1}{2}},$$

where I is the identity matrix, A is the adjacency matrix of the network with entries $a_{ij} = d_{ij}$ (the distance) if i and j are connected, 0 otherwise. D is a diagonal matrix, defined as $d_{ii} = \sum_j a_{ij}$ with $a_{ij} \in A$. Both of these normalized Laplacian matrices are used for drug target prioritization in such a way, that it diffuses differential expression values with a kernel, representing the neighborhood dependencies of the nodes within the network. In general the kernel K can be computed as follows:

$$K = \lim_{n \to \infty} (I - \frac{\alpha}{n} L)^n.$$

Laenen *et al.* showed that the best results can be obtained by using $\alpha = 0.5$ and $n = 1$, which means that the kernel only considers direct neighbors, hence it is a local measurement. In order to obtain a score vector for ranking of every gene, one has to multiply by the fold change data of all genes:

$$KDR = FC * K,$$

where FC is the vector of the fold change values of all genes corresponding to the kernel matrix K.

Correlation Diffusion Ranking

The correlation diffusion ranking is an alternative for the kernel diffusion approach, it is based on the correlation of a node to its neighbors [2]. For every pair of nodes, a Pearson correlation ρ is computed:

$$\rho_{i,j} = \frac{cov(w_i, w_j)}{\sigma_{w_i}\sigma_{w_j}},$$

where w_i is a distance vector for the node i and all other nodes, σ_{w_i} is the standard deviation of w_i. A threshold s is applied to filter the correlation elements of a normalized correlation matrix C:

$$C_{ij} = \begin{cases} \frac{\rho_{ij}}{\sum_j \rho_{ij}} & \forall \rho_{ij} \geq s, \\ 0 & \text{otherwise.} \end{cases}$$

In order to integrate the fold change data, the correlation matrix is multiplied by the FC vector, the final scores of the correlation diffusion ranking are obtained by:

$$CDR = FC * C.$$

Cytoscape Measures

The following measures are provided from Cytoscape tool (v. 2.8.3). These measures do not take the fold change data into account, only network topology is considered.

$$Eccentricity(n) = \max_{n \neq m} |sp(n,m)|,$$

where $\max$ is a function shows the maximum length of the shortest path, ignoring not reachable nodes.

$$Degree(n) = |N(n)|,$$

where N is a function returns a list of neighbors of a given node.

$$Clustering\_Coefficient(n) = \frac{e_n}{Degree(n)(Degree(n) - 1)},$$

where e_n is the number of neighbors of node n, which are connected with each other.

$$Topological\_Coefficient(n) = \frac{avg(J(n,m))}{Degree(n)},$$

where $J(n,m)$ returns the number of neighbors shared by n and m. If n and m are directly connected, 1 is added to $J(n,m)$ value.

Measure	Expression	Network	Local	Description
Local Radiality	•	•	•	SP
Fold change	•			
p-value	•			
Symmetric Kernel Diff. R.	•	•	•	RW
Correlation Diff. R.	•	•	•	RW
PeC	•	•	•	
Degree		•	•	
Clustering Coefficient		•	•	
Topological Coefficient		•	•	
Betweenness		•		SP
Radiality		•		SP
Stress		•		SP
Eccentricity		•		SP

Supplementary Table 1: Classification and description of the measures. The *Expression* column indicates the consideration of the gene expression data in the measure calculation. The *Network* column indicates the integration of the network topology. *Local* indicates local network measures. If a measure is network dependent and not local, it is called global. The *SP* and *RW* indicate the shortest path- and random walk-based method, respectively.

Statistics	FT	FT1	PT
Total # of Drugs	551	549	514
Total # of Unique Targets	2782	195	605
Avg. # of Targets per Drug	27.8	1	5.8
Stdev. # of Targets per Drug	51.3	0	18.4
Max # of Targets per Drug	403	1	222
Total # of Drug-Target Interactions	15358	1554	2676
Coverage of Targets in PPI network	93 %	96 %	90%

Supplementary Table 2: Statistics about drug-target set used in this study. Functional targets are covered in FT and FT1. Physical targets are represented in PT. All datasets contain around 500 drugs that have at least one known target. FT is the most comprehensive functional target set in terms of total number of targets and drug-target interactions. On average 93% of all target proteins are represented in the protein-protein interaction network.

	Betweenness	Clustering Coef.	Correlation Diff. R.	Degree	Eccentricity	P-value	FC-value	Sym. Kernel Diff. R.	PeC	Radiality	Local Radiality	Stress	Topological Coef.
Betweenness	1	3,77e-53	1,02e-53	2,69e-39	4,38e-51	6,21e-30	2,39e-41	0,2828	9,79e-06	1	1	1	0,0422
Clustering Coef.	1	1	0,3997	1	1	1	1	1	1	1	1	1	1
Correlation Diff. R.	1	0,6046	1	1	1	1	1	1	1	1	1	1	1
Degree	1	5,53e-47	2,91e-47	1	3,46e-43	7,84e-10	1,09e-21	1	1	1	1	1	1
Eccentricity	1	9,38e-21	2,15e-23	1	1	1	1	1	1	1	1	1	1
P-value	1	3,09e-36	7,70e-37	0,9999	4,02e-18	1	5,96e-05	1	1	1	1	1	1
FC-value	1	2,14e-28	1,70e-29	1	2,33e-10	0,9999	1	1	1	1	1	1	1
Sym. Kernel Diff. R.	0,7176	1,28e-52	4,54e-53	1,79e-36	1,36e-50	6,00e-30	5,50e-42	1	1,53e-05	1	1	0,9999	0,1710
PeC	0,9999	5,44e-50	5,80e-50	1,19e-18	3,26e-43	3,00e-24	1,73e-36	0,9999	1	1	1	1	0,9974
Radiality	1,48e-16	3,35e-55	1,61e-55	4,31e-43	3,27e-53	9,09e-35	6,25e-45	2,66e-12	1,11e-22	1	0,9999	0,0619	1,91e-19
Local Radiality	2,77e-16	4,65e-57	8,34e-57	1,26e-44	1,90e-55	4,18e-38	1,61e-48	1,79e-16	5,73e-24	6,28e-05	1	1,14e-06	3,01e-17
Stress	2,98e-12	4,53e-55	2,18e-55	1,55e-44	6,89e-53	5,57e-35	3,28e-46	1,42e-08	6,51e-16	0,9382	0,9999	1	5,42e-10
Topological Coef.	0,9578	2,01e-53	3,60e-53	7,90e-34	4,40e-51	3,17e-29	1,58e-40	0,8293	0,0025	1	1	0,9999	1

Supplementary Table 3: Statistical significance of the recall curves for the FT set (given in **Supplementary Fig. 2**a). Two groups Wilcoxon signed rank was applied to show how significant a recall curve of a measure compared to another one. Note that: the p-value in a cell *(i,j)* indicates significance of the measure in the ith row compared to the measure in the jth column.

	Betweenness	Clustering Coef.	Correlation Diff. R.	Degree	Eccentricity	P-value	FC-value	Sym. Kernel Diff. R.	PeC	Radiality	Local Radiality	Stress	Topological Coef.
Betweenness	1	1,61e-43	6,19e-39	3,70e-12	1,57e-36	9,21e-32	1,45e-35	0,0913	2,00e-05	0,9979	0,9805	0,9403	0,0314
Clustering Coef.	1	1	1	1	0,9999	0,9999	0,9914	1	1	1	1	1	1
Correlation Diff. R.	1	1	1	1	0,9999	0,9999	0,9914	1	1	1	1	1	1
Degree	1	2,98e-33	2,08e-30	1	3,01e-26	1,86e-21	2,04e-26	0,9999	0,7114	1	1	1	0,9999
Eccentricity	1	8,20e-09	3,10e-07	1	1	0,3815	5,26e-05	1	1	1	1	1	1
P-value	1	8,13e-07	3,89e-06	1	0,6241	1	2,86e-05	1	1	1	1	1	1
FC-value	1	0,0184	0,0184	1	0,9999	0,9999	1	1	1	1	1	1	1
Sym. Kernel Diff. R.	0,9102	8,59e-37	1,05e-33	5,87e-06	3,39e-29	6,54e-27	1,36e-31	1	0,0026	0,9978	0,9984	0,9859	0,5595
PeC	0,9999	1,45e-30	3,11e-28	0,2895	1,25e-19	2,56e-18	2,84e-24	0,9974	1	0,9999	0,9999	0,9999	0,9984
Radiality	0,0024	6,38e-46	4,05e-41	1,36e-14	6,19e-39	2,62e-34	9,60e-38	0,0021	4,56e-08	1	0,7714	0,0823	4,28e-06
Local Radiality	0,0197	1,16e-45	4,12e-41	4,98e-11	1,81e-38	3,41e-33	3,17e-37	0,0015	3,42e- 06	0,2301	1	0,0692	0,0012
Stress	0,0725	1,30e-44	8,28e-40	2,88e-13	1,27e-37	5,30e-33	1,95e-36	0,0144	2,17e-06	0,9249	0,9313	1	0,0054
Topological Coef.	0,9708	1,48e-41	5,73e-37	1,04e-06	1,45e-34	8,29e-30	1,32e-33	0,4438	0,0015	0,9999	0,9988	0,9948	1

Supplementary Table 4: Statistical significance of the recall curves for the FT1 set (given in **Supplementary Fig. 2**b).

	Betweenness	Clustering Coef.	Correlation Diff. R.	Degree	Eccentricity	P-value	FC-value	Sym. Kernel Diff. R.	PeC	Radiality	Local Radiality	Stress	Topological Coef.
Betweenness	1	1,50e-14	2,35e-15	3,70e-10	2,35e-15	2,92e-05	5,09e-14	0,9985	0,9362	0,9999	0,9999	0,9944	0,9999
Clustering Coef.	1	1	0,5	0,9999	0,1855	1	0,9901	1	1	1	1	1	1
Correlation Diff. R.	1	0,9772	1	1	1	1	0,9993	1	1	1	1	1	1
Degree	1	1,02e-09	1,15e-10	1	1,15e-10	0,4398	3,11e-08	1	1	1	1	1	1
Eccentricity	1	0,9631	1	1	1	1	0,9993	1	1	1	1	1	1
P-value	0,9999	3,52e-10	3,66e-11	0,5620	3,66e-11	1	9,56e-09	0,9999	0,9999	0,9999	0,9999	0,9999	0,9999
FC-value	1	0,0115	0,0008	0,9999	0,0008	0,9999	1	1	1	1	1	1	1
Sym. Kernel Diff. R.	0,0014	3,54e-17	3,54e-17	5,24e-12	3,54e-17	1,27e-07	7,02e-16	1	0,4157	0,9483	0,9901	0,0220	0,7404
PeC	0,0646	3,60e-16	3,60e-16	6,76e-11	3,60e-16	9,95e-07	1,12e-14	0,5867	1	0,9186	0,9899	0,1295	0,7808
Radiality	3,50e-06	2,61e-17	4,85e-18	7,23e-13	4,85e-18	1,14e-08	5,95e-17	0,0531	0,0824	1	0,9081	0,0004	0,0044
Local Radiality	7,86e-06	3,89e-18	7,34e-19	1,99e-13	7,34e-19	1,26e-09	8,45e-18	0,0101	0,0101	0,0943	1	7,95e-05	0,0297
Stress	0,0060	1,70e-15	2,41e-16	2,52e-11	2,41e-16	8,49e-06	7,59e-15	0,9788	0,8718	0,9995	0,9999	1	0,9961
Topological Coef.	3,71e-05	1,68e-16	3,28e-17	4,87e-12	3,28e-17	6,43e-08	4,06e-16	0,2646	0,2210	0,9968	0,9711	0,0042	1

Supplementary Table 5: Statistical significance of the recall curves for the PT set (given in **Supplementary Fig. 2**c).

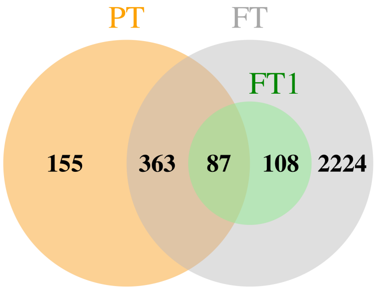

Supplementary Figure 1: The overlap of unique targets in three target data sets. 87 known targets are provided by all drug-target data sets. However, 155 physical drug-targets are covered by only PT. 2224 known targets are only found in FT.

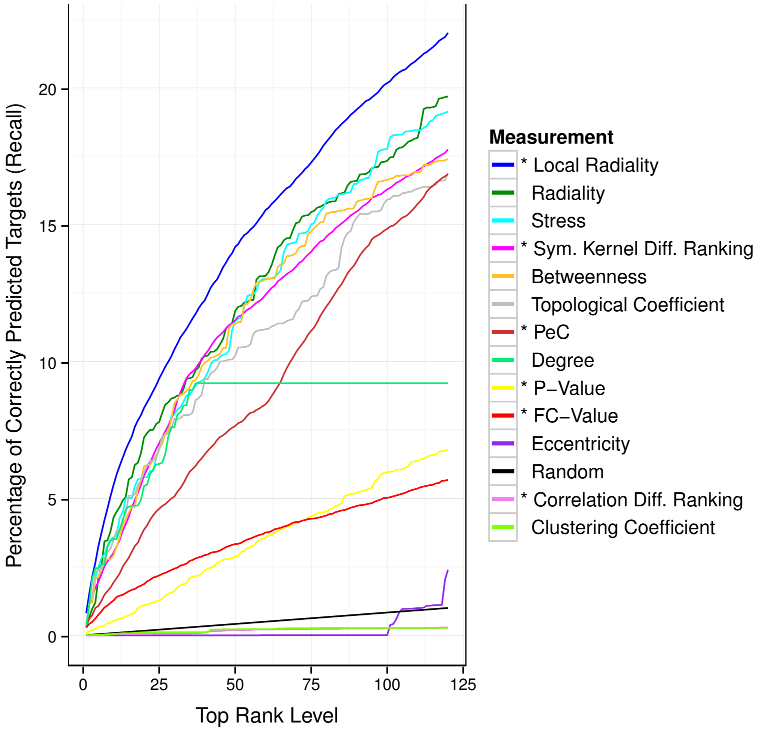

(a) FT

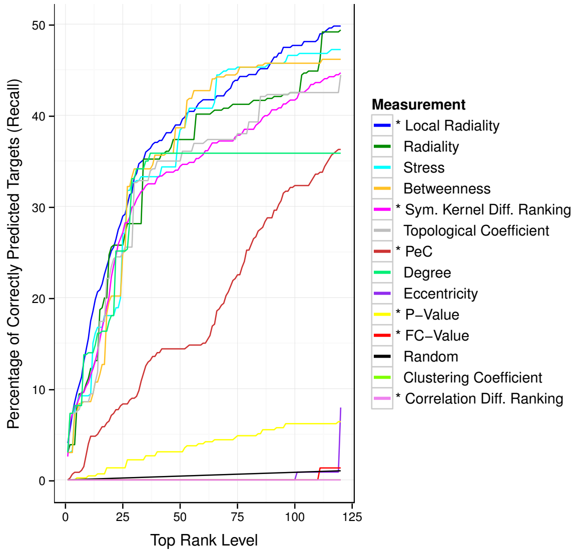

(b) FT1

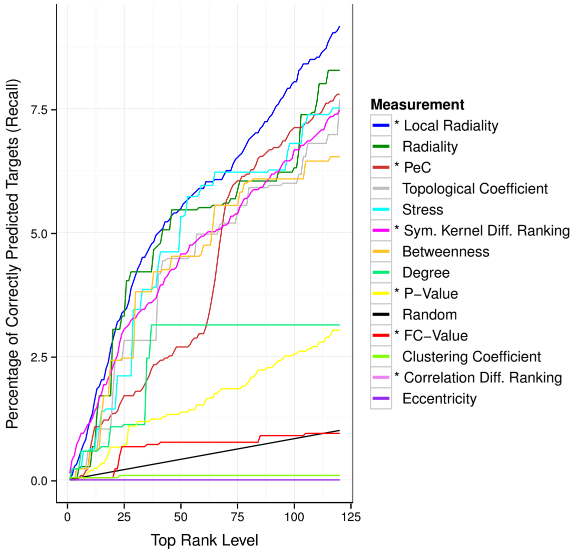

(c) PT

Supplementary Figure 2: Systematic comparison of all measures from **Supplementary Table 1**. For the functional targets (FT, FT1), local radiality, stress and radiality measures achieved the highest performance. Local radiality, radiality and PeC are the best measures for the physical targets (PT). The measures are ordered by their prediction performance in the 1st percentile. Measurements with * take fold change data into account.

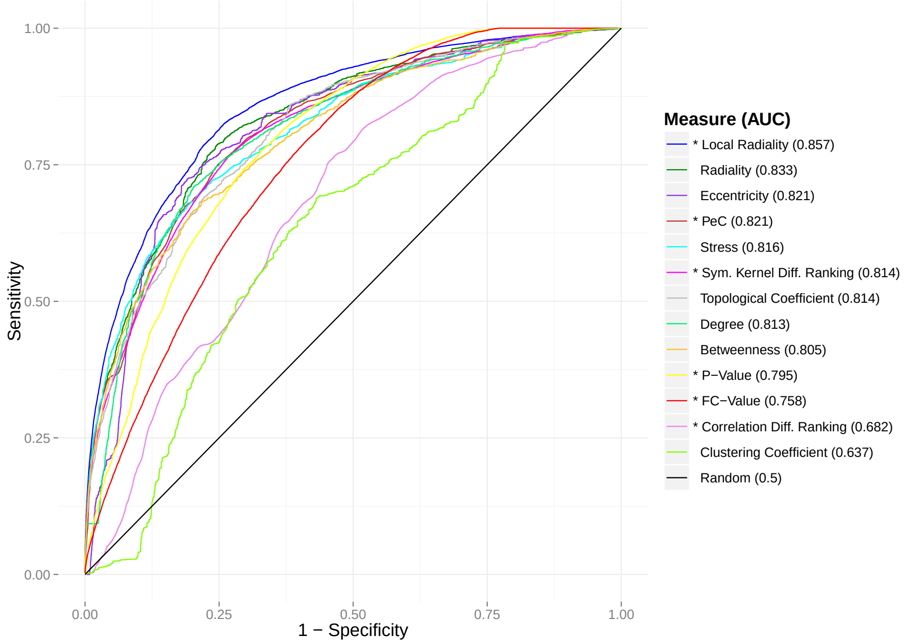

(a) FT

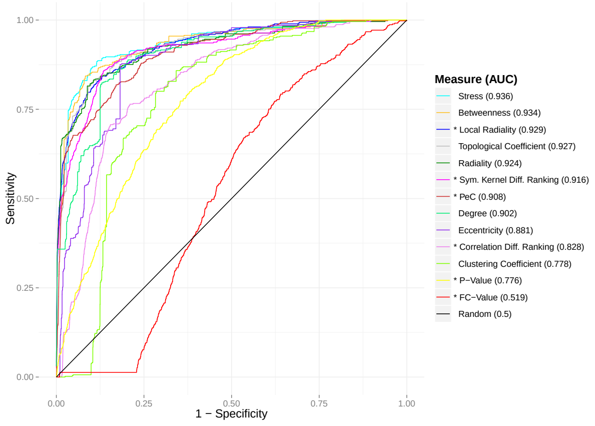

(b) FT1

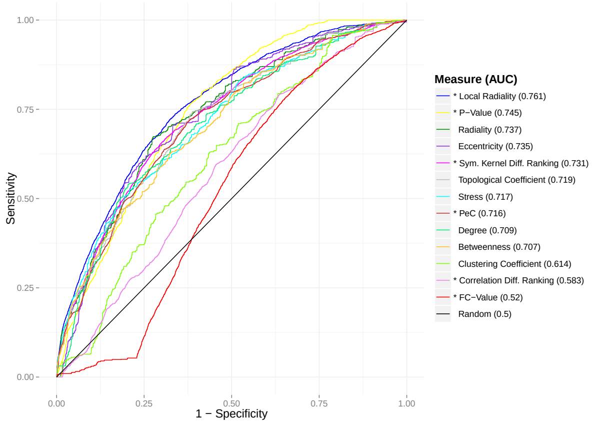

(c) PT

Supplementary Figure 3: ROC curves of all measures from **Supplementary Table 1**. Local radiality, radiality and eccentricity achieved the highest AUC values for the functional target FT set. However, stress, betweenness and local radiality have very close AUC values for FT1 set. Local radiality, gene expression p-value and radiality are the best measures for the physical targets (PT). Measurements with * take fold change data into account.

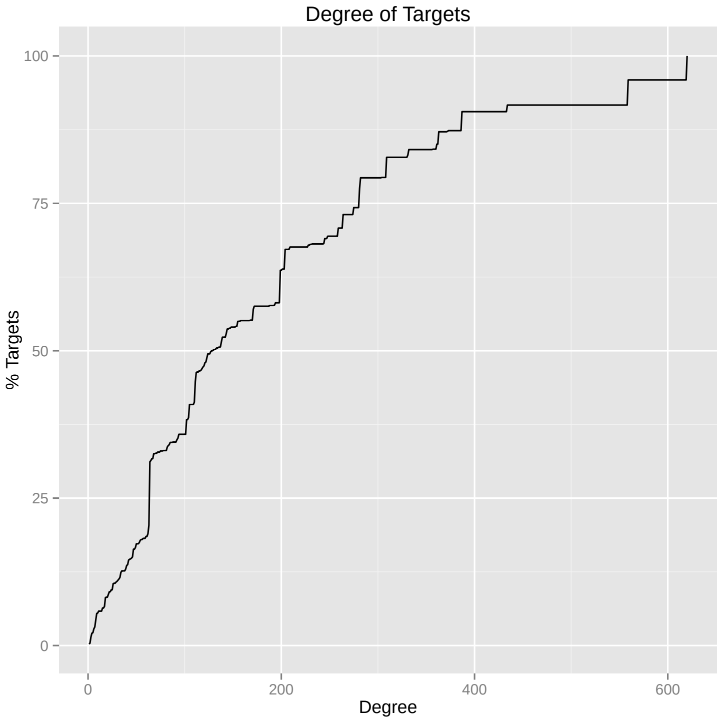

Supplementary Figure 4: The distribution of targets according to the degree. More than 82% of functional targets (FT1) have more than 50 neighbors in the STRING network.

References

[1] M. Li, H. Zhang, J. xin Wang, and Y. Pan, “A new essential protein discovery method based on the integration of protein-protein interaction and gene expression data.,” *BMC Syst Biol*, vol. 6, p. 15, 2012.

[2] G. Laenen, L. Thorrez, D. Bornigen, and Y. Moreau, “Finding the targets of a drug by integration of gene expression data with a protein interaction network,” *Mol. BioSyst.*, 2013.
